# Endoluminal vacuum therapy in the management of an esophago-pleural fistula as a complication of Boerhaave syndrome in a patient with eosinophilic esophagitis

**DOI:** 10.1186/s12876-021-02058-z

**Published:** 2021-12-20

**Authors:** Carlos Tuñon, Juan De Lucas, Jan Cubilla, Rafael Andrade, Miguel Aguirre, Julio Zúñiga Cisneros

**Affiliations:** 1 Department of Gastroenterology, Santo Tomas Hospital, Panama City, Panama; 2 Department of Thoracic Surgery, Santo Tomas Hospital, Panama City, Panama; 3 Department of Internal Medicine, Santo Tomas Hospital, Bella Vista, Panama City, Panama

**Keywords:** Boerhaave syndrome, Eosinophilic esophagitis, Vacuum-assisted closure, Endoscopy, Case report

## Abstract

**Background:**

Boerhaave syndrome is an uncommon condition that represents about 15% of all esophageal perforation. A subset of these patients has eosinophilic esophagitis, a chronic inflammatory disease of the esophagus, that carries a risk of perforation of about 2%. Esophageal perforations can rarely result in the development of an esophago-pleural fistula. Treatment of esophago-pleural fistula represent a challenge due to lack of high quality evidence and scarce reported experience. Endoluminal vacuum-assisted therapy could have a role in the management by using the same principle applied in external wounds which provide wound drainage and tissue granulation.

**Case presentation:**

We report a unique case of a 24-year-old man with eosinophilic esophagitis complicated with an esophageal rupture who developed an esophago-pleural fistula and was successfully managed with a non-surgical approach using endoluminal vacuum-assisted therapy. To our knowledge this could be the first experience reported in a patient with eosinophilic esophagitis.

**Conclusion:**

Endoluminal vacuum-assisted therapy might be an effective and novel strategy in patients with eosinophilic esophagitis and esophago-pleural fistula as a consequence of Boerhaave syndrome. Appropriately designed studies are required.

## Background

Effort rupture of the esophagus, also known as Boerhaave syndrome is uncommon. It represents about 15% of all esophageal perforation which have an incidence of 3.1 per 1,000,000 per year [1, 2]. A subset of patients with Boerhaave syndrome have underlying eosinophilic esophagitis. On the other hand, approximately 2% of patients with eosinophilic esophagitis can develop an effort rupture of the esophagus [3]. Amid the complications of Boerhave syndrome, esophago-pleural fistula is uncommon despite the anatomical proximity of these structures [2].

The mortality of Boaerhaave syndrome can be 10% if diagnosed within less than 24 h of symptoms and can be as high as 50% if diagnosed late. Management of esophageal perforation depends on the size, location, and complications associated with perforation; as well as the comorbidities of the patient and the baseline status of the esophagus [2, 4]. Surgery is the mainstay of treatment, but lately nonoperative management has been the preferred approach in a substantial number of patients. Non-operative treatment options include conservative esophageal stenting and endo-clip application. However, their routine use in the context of an esophago-pleural fistula have not been established [4].

During the last years, there has been some reports the role of endoluminal vacuum therapy in the management of esophageal perforations and leaks with acceptable high success rates of up to 83%. Nonetheless, the experience in the setting of Boerhaave syndrome and esophago-pleural fistula is limited [5, 6]. There are no reported cases in eosinophilic esophagitis.

We report the case of a patient with eosinophilic esophagitis that was complicated with an esophageal perforation developing esophago-pleural fistula and was managed successfully with endoscopy vacuum-assisted closure with sponge.

## Case presentation

A 24-year-old man was admitted to our hospital 48 h after developing an acute retrosternal chest pain that was radiated to the upper back, associated with multiple episodes of vomit (food content), and progressive dyspnea. Additionally, he describes intermittent difficulty swallowing solid food. His medical history is significant for well-controlled asthma using salbutamol as needed.

Initial evaluation revealed a temperature of 38.4 degrees; blood pressure, 100/60 mmHg; heart rate, 118/min; respiratory rate, 26/min, and SpO_2_, 94% on room air. Additionally, the physical examination showed subcutaneous emphysema in the cervical and thoracic regions, shallow breathing, dullness to percussion in both lung bases. His investigation results on admission revealed white blood cell count of 21,000/μL (91% Neutrophils), C-reactive protein (CRP) and procalcitonin were 39.2 mg/dL and 9 ng/mL respectively. Liver and renal function were normal. The patient was admitted with sepsis of unclear etiology, although there was a suspicious for esophageal perforation predisposing mediastinitis and sepsis.

**Fig. 1:**
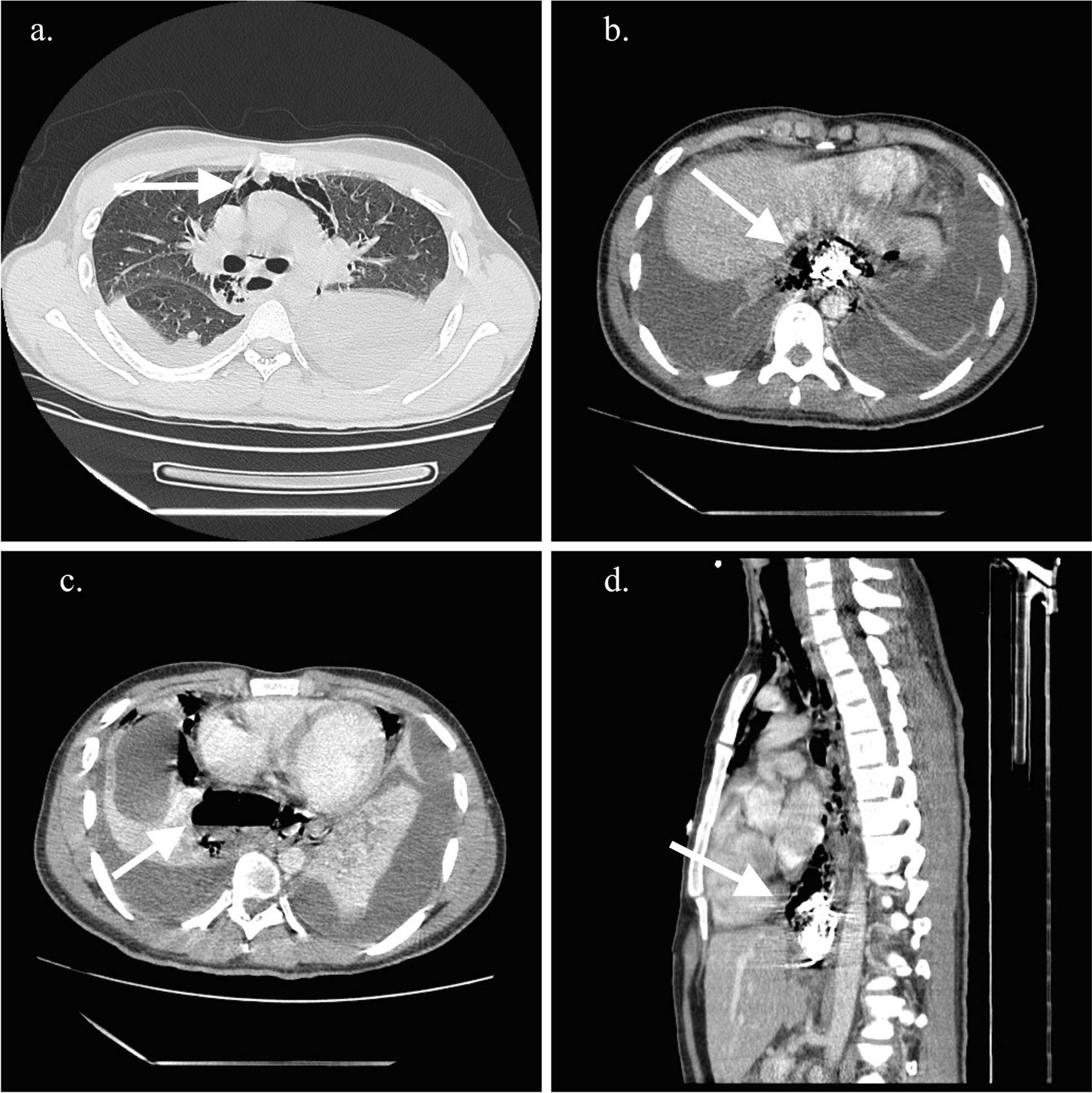
Contrast-enhanced CT findings. **a** CT findings of pneumomediastinum (white arrow), **b** extraluminal residual of oral contrast, **c** Fluid-air level in distal esophagus, **d** extraluminal oral contrast on perforation area (white arrow)

**Fig. 2:**
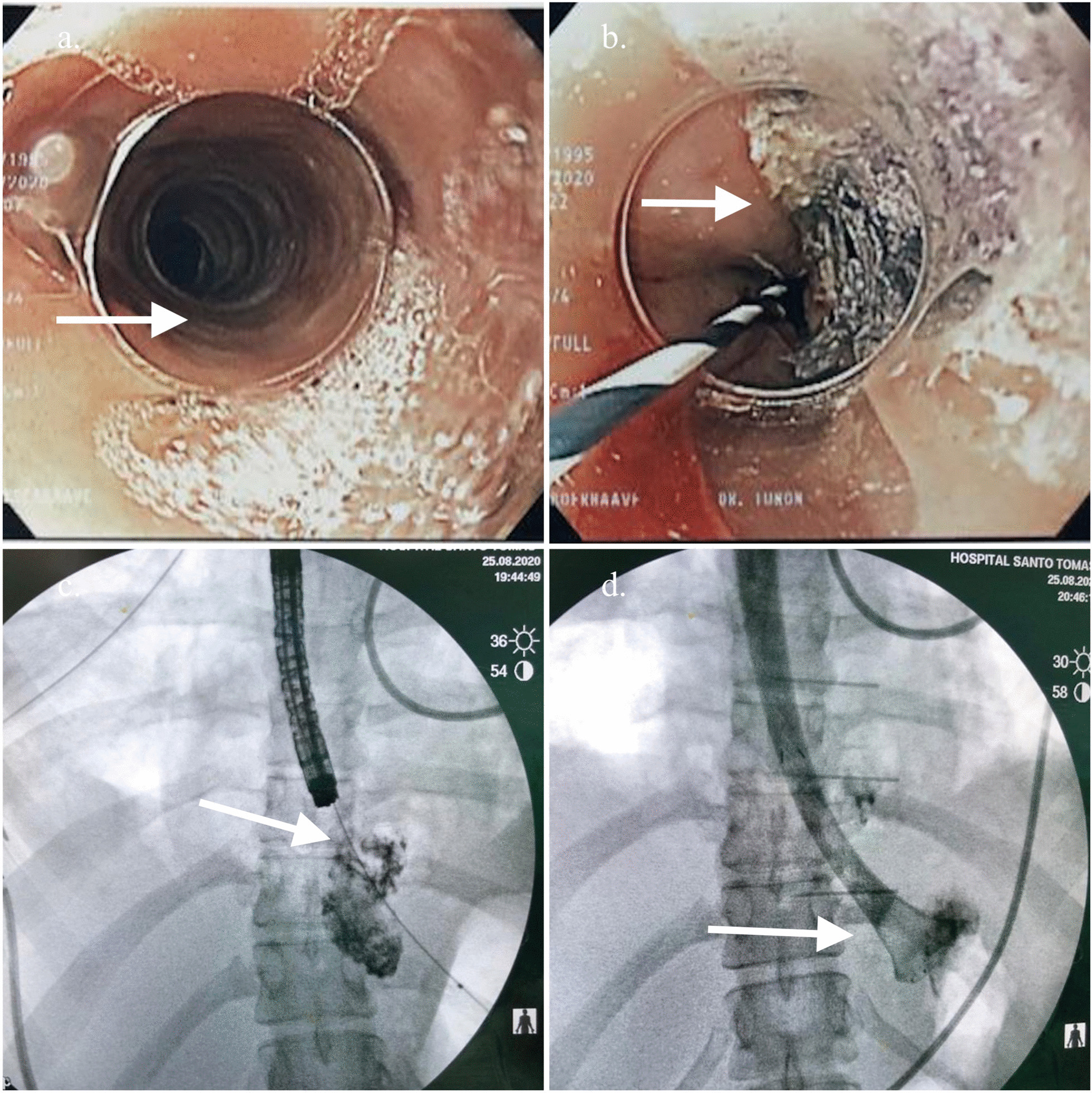
Intraoperative endoscopy. **a** Typical endoscopic findings of eosinophilic esophagitis, circular rings and linear furrows. **b** Narrow banding imaging with endoscopy showing the esophageal tear (with different color). **c**. Endoscopy vision of esophageal perforation area with residual oral contrast and detritus. **d** fluoroscopy showing extraluminal residual oral contrast

A contrast-enhanced thoracic computed tomography (CT), showed a pneumomediastinum, cervical emphysema, bilateral pleural effusion, as well as extraluminal oral contrast surrounding the distal portion of the gastro-esophageal junction region and fluid-air level indicating a collection in the posterior mediastinum (Fig. 1a–d). Initial management included intravenous fluid, nothing per oral (NPO), broad spectrum antibiotics, and analgesia. Due to the clinical condition of the patient, time of the rupture and inaccessibility to an intensive care unit due to the Covid 19 pandemic situation, the thoracic surgery and gastroenterology teams decided a nonoperative approach based on endoscopic therapy. The patient underwent endoscopy that showed a distal esophageal lineal tear just above Z line of approximately 4 cm with irregular edges. Irrigation and drainage of food debris of the cavity was performed before a distal auto-expandable esophageal prosthesis SX-ELLA (ELLA-CS) of 25 mm × 18 mm × 15 cm with antimigration technology and anti-reflux valve was placed (Fig. 2). The thoracic surgeon decided to put a bilateral pleural tube oriented toward the perforation preventing future complications. Biopsies of the esophageal mucosa were obtained confirming the clinical suspicion of eosinophilic esophagitis (Fig. 2a).

### Esophago-pleural Fistula and endoluminal vacuum-assisted closure with sponge

**Fig. 3:**
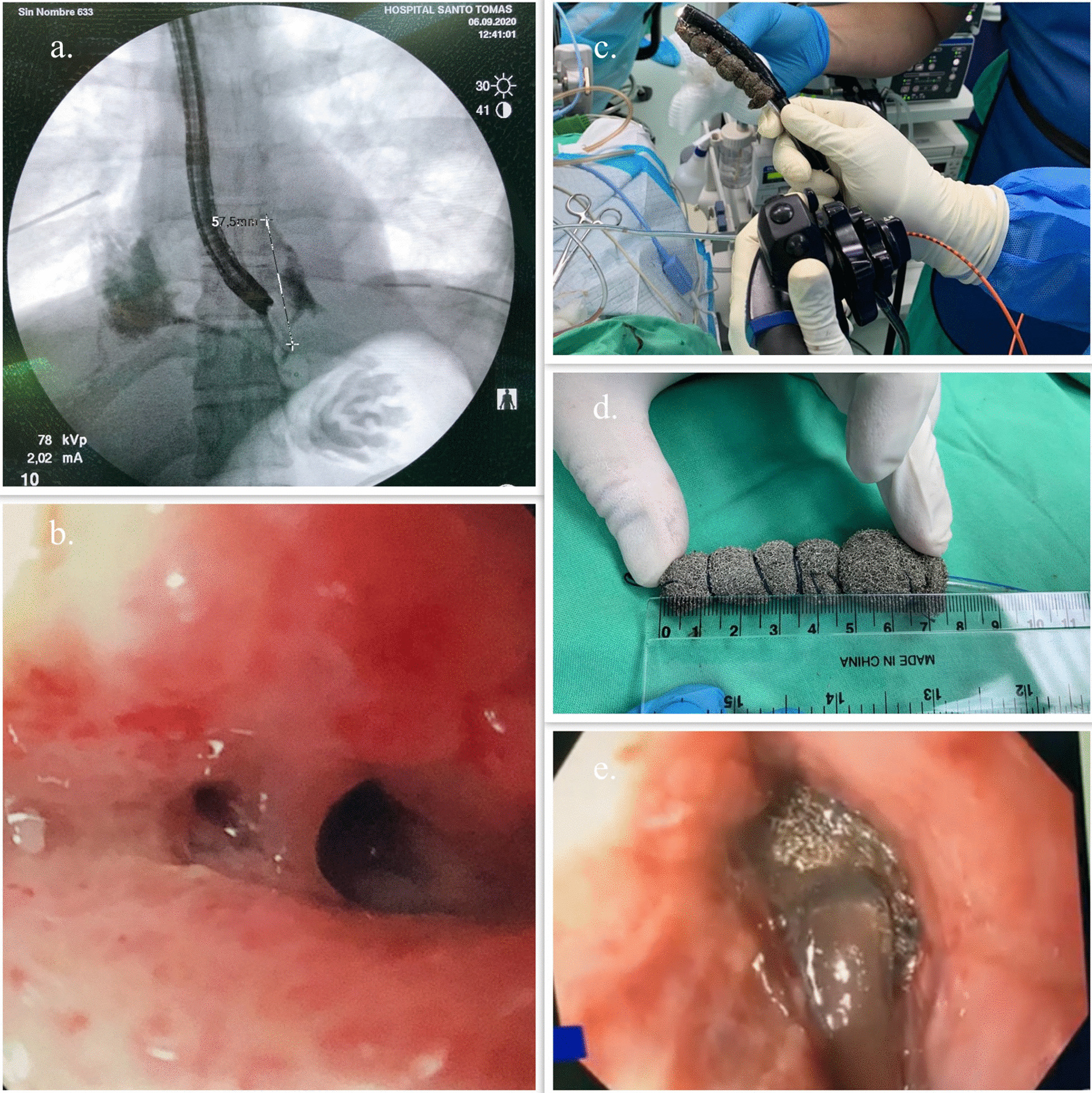
Management of the Esophageal-pleural fistula. **a** Fistulogram showing an esophageal-pleural fistula. **b** Endoscopy finding in distal esophagus. **c**–**e** endoscopy vacuum-assisted closure with sponge for esophago-pleural fistula

Five days after admission, a new endoscopic procedure was performed, showing migration of the esophageal stent into the stomach, a persistent perforation defect in the lower third of the esophagus with granulation tissue and two small cavities suggesting a fistulous tract. A fistulogram was performed confirming the fistulous tract between the esophagus and the pleura (Fig. 3a, b). Based on these findings and previous case reports found in the literature it was decided to place an endoluminal vacuum-assisted closure with sponge in the area of perforation with the fistula (Fig. 3c–e) to control both complications. The sponge was cutted to 7 cm, adjusted and grasped with a tripod equipped endoscope and introduced in the cavity under direct visualization. After placement of the sponge, a vacuum device was connected and set to a continuous 125 mmHg sub-atmospheric, moderate intensity pressure.

**Fig. 4:**
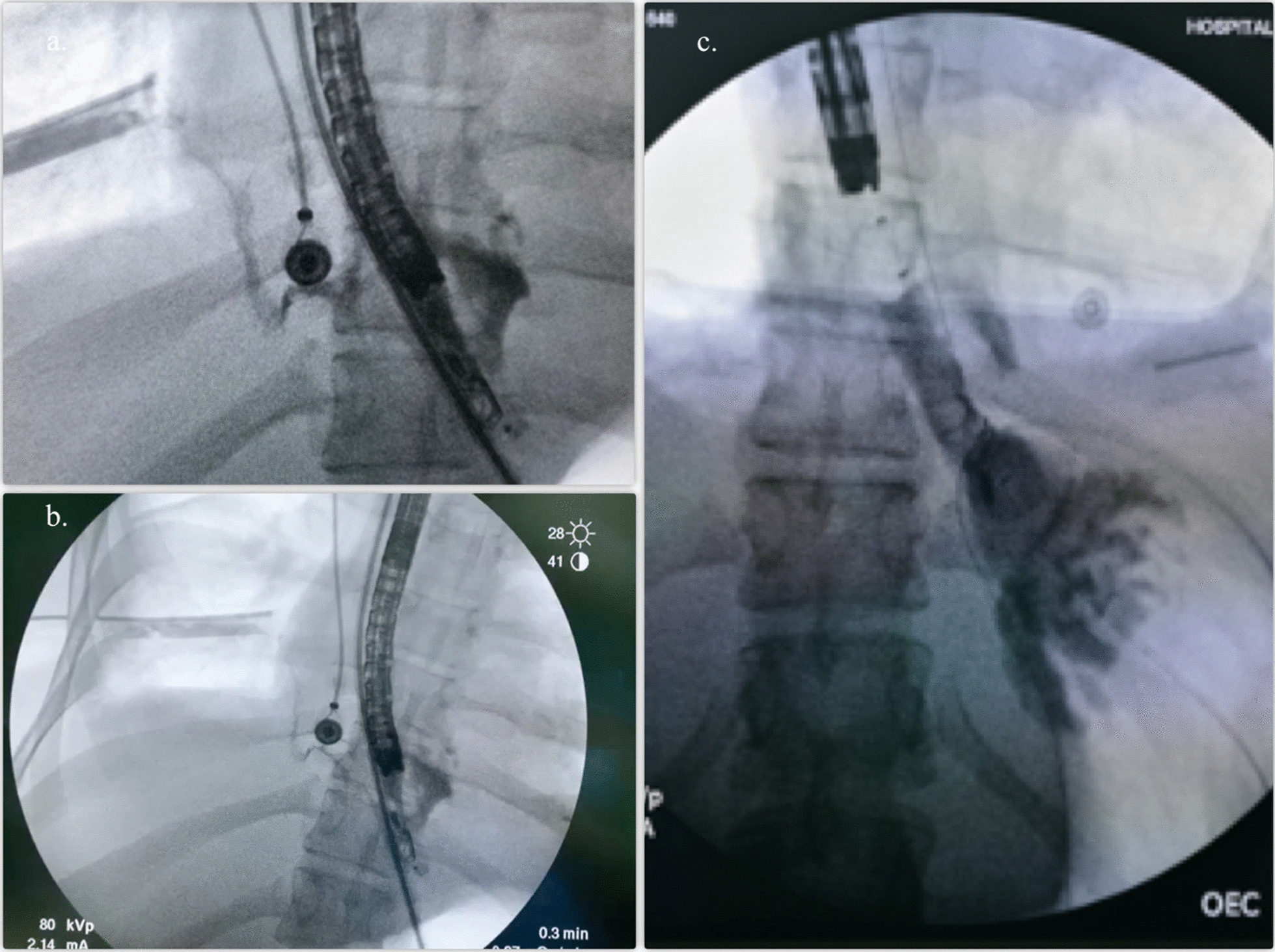
**a**, **b** Esophageal-pleural fistula evolution after six (6) dressing change of endoscopic vacuum-assisted closure with sponge. **c** Fistulogram showing resolution of the esophago-pleural fistula in distal esophagus with esophageal balloon anchored in both sides

In the second intervention for dressing change of the sponge, two OVESCO clips (OTSC®) were placed as a strategy to reduce the size of the tear and closure of the fistula, reducing up to 30% of the longitudinal size. The patient required four additional dressing changes of endoscopic vacuum-assisted closure with sponge, each one performed every 72 h, until the fistulogram showed resolution of the esophago-pleural fistulous tract (Fig. 4a–c).

Despite good progress of the esophago-pleural fistula, the patient condition got worse due *Clostridium difficile* colitis. Despite appropriate antibiotic treatment, and resucitacion with fluids the patient developed shock with severe acute respiratory distress syndrome requiring vasopressor support and mechanical ventilation with neuromuscular blockade. The gastroenterology team decided to place a new esophageal prothesis in order to avoid dressing changes of the sponge and worsening of the clinical condition. Considering *Clostridium difficile* infection, a recto-sigmoidoscopy was performed which reported ischemic colitis and pseudomembranes. Due to lack of improvement despite treatment due to uncontrolled foci of infectious (colon), an emergency left hemicolectomy and a Hartmann’s procedure were performed.

**Fig. 5:**
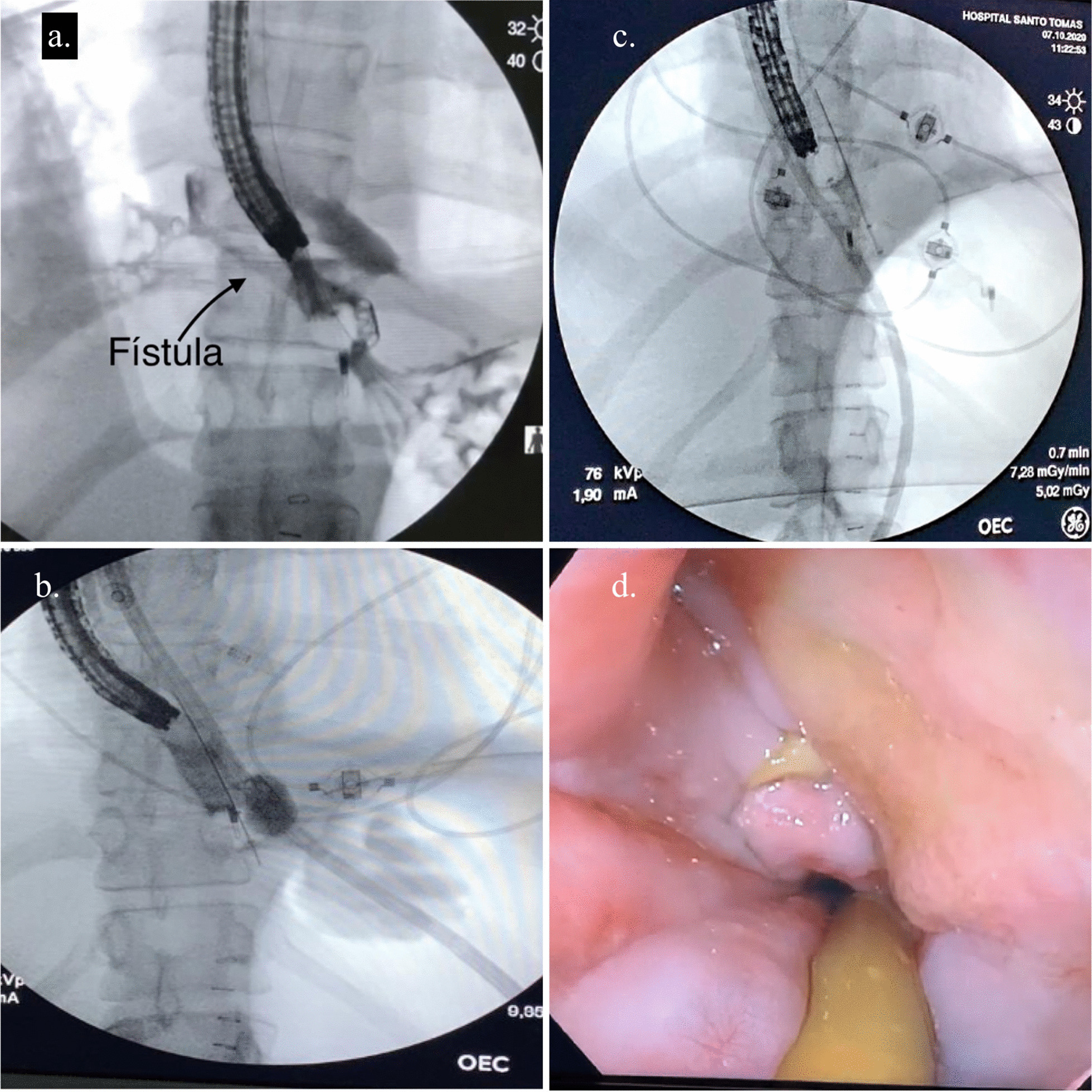
**a** Fistulogram after second esophageal prothesis showing relapsing sophago- pleural fistula. **b** and **c** Fistulogram showing resolution of the esophago-pleural fistula in distal esophagus after 3 dressing change of endoscopic vacuum-assisted closure with sponge. **d** Endoscopy showing area of distal rupture epithelized and healed

Over the next 7 days, the patient condition improved, allowing to perform an endoscopy with fistulogram that showed a recurrent fistulous esophago-pleural tract (Fig. 5a). The esophageal prothesis was removed and a new vacuum-assisted closure with sponge was placed. Three dressing changes of endoscopic vacuum-assisted closure with sponge were performed before esophago-pleural fistula resolution was evident (Fig. 5b, c). The sponge and the OTSC® was retired due to a complete fistula resolution.

A follow-up endoscopy performed 3 days after the last vacuum-assisted closure with sponge was removed, that showed epithelized esophageal mucosa with granulation tissue (Fig. 5d). After 46 days the patient was discharged with outpatient follow-up.

Two months later, the patient was asymptomatic, tolerating solids in the diet. A prednisone base therapy and food elimination diet were initiated as a measure of control of his eosinophilic esophagitis.

## Discussion and conclusion

To our knowledge, the case presented here could be the first case reported of eosinophilic esophagitis and esophago-pleural fistula secondary to Boerhaave syndrome managed successfully with endoluminal vacuum-assisted closure with sponge.

The best management strategy for esophageal perforation and esophago-pleural fistula, regardless of the cause, is still controversial due to the lack of appropriate clinical studies comparing interventions. In addition, there is scarce information about esophago-pleural fistula and Boerhaave syndrome in patients with eosinophilic esophagitis [2, 6].

Endoluminal vacuum therapy has become an alternative endoscopic strategy in patients with contraindications for surgery. It can be used in critically ill and hemodynamically unstable patients that need source control, allowing removal of necrotic debris while promoting tissue healing and granulation tissue in the first 3–5 days of therapy [6–8]. Our patient was managed with a non-surgical strategy given his critical condition and time of presentation. Initially, the defect was managed unsuccessfully with a distal auto-expandable esophageal prosthesis; however, due to migration of the prosthesis and development of an esophago-pleural fistula the gastroenterologist chose an endoluminal vacuum therapy as a possible therapy based on previous reports of successfully treated patients [8–10]. There are no current guidelines defining the indications for vacuum therapy in the setting of esophageal rupture and its complications. Although two successful cases in a postoperative esophageal anastomotic leak and bronchogenic cyst communicating with the esophagus has been reported [9].

In our case we decided a novel approach considering the size of the rupture and the development of a fistulous tract with an off-label use of two OVESCO clip (OTSC®) to minimize the defect; however, it was not succesful. In the literature we found a case reporting healing of a fistulous tract in a patient with a traumatic esophago-pleural fistula using Over-the-Scope-Clip System with good outcome, although in our case the inflammation and eosinophilic infiltration of the esophageal tissue could have played an important setback for success due to persistent inflammation [11].

There are reports describing the beneficial effects of vacuum therapy to promote healing effect; based on different mechanism included macro and microdeformation, changes in perfusion, exudate control, and bacterial clearance [6, 8]. These mechanisms might have had an impact in our patient, unlike other treatment options initially used. We hope the successful outcome of this case offers a new treatment alterative in this scenario. As a single case experience, more studies are required to validate our observation and establish a true efficacy, however we think it could open new avenues for future research.

This case suggests that an endoluminal vacuum sponge therapy with negative pressure might be an effective and novel strategy in patient with eosinophilic esophagitis who developed esophago-pleural fistula as a consequence of Boerhaave Syndrome. We hypothesize that in the context of Boerhaave syndrome in patients with eosinophilic esophagitis, new strategies such as the vaccum therapy could provide another resource for managing these patients.

## Data Availability

All data generated or analyzed during this case report are included in this published article.

## Abbreviations

SpO_2_: Oxygen saturation
CRP: C reactive protein
CT: Computed tomography
